# Interprofessional teamwork in the trauma setting: a scoping review

**DOI:** 10.1186/1478-4491-11-57

**Published:** 2013-11-05

**Authors:** Molly Courtenay, Susan Nancarrow, David Dawson

**Affiliations:** 1 School of Health and Social Care, Faculty of Health and Medical Sciences, University of Surrey, Guildford, Surrey, UK; 2 University of California, Davis, Betty Irene Moore School of Nursing, UC Davis Health System, 4610 X Street, #4202, Sacramento, CA 95817, USA; 3 Southern Cross University, School of Health and Human Sciences, Lismore, Australia; 4 Lawrence J. Ellison Ambulatory Care Center, University of California, Davis, Sacramento, CA, USA

**Keywords:** Interprofessional teamworking, Interprofessional collaborative practice, Trauma setting

## Abstract

Approximately 70 to 80% of healthcare errors are due to poor team communication and understanding. High-risk environments such as the trauma setting (which covers a broad spectrum of departments in acute services) are where the majority of these errors occur. Despite the emphasis on interprofessional collaborative practice and patient safety, interprofessional teamworking in the trauma setting has received little attention. This paper presents the findings of a scoping review designed to identify the extent and nature of this literature in this setting. The MEDLINE (via OVID, using keywords and MeSH in OVID), and PubMed (via NCBI using MeSH), and CINAHL databases were searched from January 2000 to April 2013 for results of interprofessional teamworking in the trauma setting. A hand search was conducted by reviewing the reference lists of relevant articles. In total, 24 published articles were identified for inclusion in the review. Studies could be categorized into three main areas, and within each area were a number of themes: 1) descriptions of the organization of trauma teams (themes included interaction between team members, and leadership); 2) descriptions of team composition and structure (themes included maintaining team stability and core team members); and 3) evaluation of team work interventions (themes included activities in practice and activities in the classroom setting).

Descriptive studies highlighted the fluid nature of team processes, the shared mental models, and the need for teamwork and communication. Evaluative studies placed a greater emphasis on specialized roles and individual tasks and activities. This reflects a multiprofessional as opposed to an interprofessional model of teamwork. Some of the characteristics of high-performing interprofessional teams described in this review are also evident in effective teams in the community rehabilitation and intermediate care setting. These characteristics may well be pertinent to other settings, and so provide a useful foundation for future investigations.

## Introduction

The importance of interdisciplinary teams in ensuring effective primary healthcare was recognized as far back as 1978 by the World Health Organization (WHO) [1]. Over a decade ago, two separate reports, each published by the Institute of Medicine (IOM), focused on the importance of collaborative practice and interdisciplinary education in healthcare. The book *Crossing the Quality Chasm: A New health System for the 21st Century*[2] emphasized the importance of collaboration and interdisciplinary training in the effective coordination of care. Patient safety and collaboration across disciplines was highlighted in a further book, *To Err is Human: Building a Safer Health System*[3]*.*

Interprofessional teamwork is achieved through interactive effort between all the professionals involved, with good communication and respect for and understanding of the roles of other team members [4]. Everyone involved in the process takes the contribution of everyone else into consideration [5]. Factors that influence interprofessional team performance include the size and psychological composition of the group (group structure), what happens when the group works together (group processes or dynamics) and how the group is led (for example by the team leader or supervisor) [6]. These ‘non-technical’ skills are of major relevance to patient safety [6,7].

In the 1980s, the UK Department of Defence developed crew resource management (CRM), to increase the safety of air operations in the military. In the USA, CRM has been integrated in all branches of the military and in commercial aviation [8]. CRM has also been adapted for use within healthcare teams in a number of settings. However, despite the emphasis on team training and the implementation of team behavior [2,3] over the past decade and, more recently, the well-documented benefits of interprofessional education and interprofessional collaborative practice [9], communication failure between healthcare team members remains a frequent cause of patient harm [10]. It is estimated that approximately 70 to 80% of healthcare errors are due to poor team communication and understanding [7] and these errors can lead to negative health outcomes, and reduced quality and safety of care [11]. High-risk environments such as the trauma setting, which involves the management of complex patients by specialized teams in a dynamic environment, and where communication, cooperation and coordination are vital for effective care, is where the vast majority of these errors occur. For example, within the UK, an average of 11 people per day are seriously harmed during surgery, and major surgical errors have risen by 28% over the past 5 years [12]. Lingard *et al*. [13] found that communication failure in the operating room (OR) occurred in around 30% of exchanges between team members, and that a third of these exchanges jeopardized patient safety.

Although the concept of interprofessional teamworking has received much attention in arenas such as primary care, rehabilitation, and geriatrics [14], less attention has focused upon this concept in highly dynamic, intense, and uncertain environments such as the trauma setting [15]. A broad spectrum of departments and settings manage trauma in acute services (including orthopedic trauma, surgery, urology, intensive care, and accident and emergency) [16]. Each of these settings influences the team structure and roles. Here, we present the findings of a scoping review designed to identify the extent and nature of the literature on interprofessional teamworking across these settings.

Scoping reviews are being used increasingly by researchers to review health research evidence [17,18], enable the clarification of complex concepts, and refine subsequent research enquiries [19]. Such reviews are particularly relevant in areas in which evidence is emerging and the paucity of randomized controlled trials (RCTs) makes it difficult for researchers to undertake systematic reviews [20]. Researchers are able to incorporate a range of study designs that address questions beyond those related to the effectiveness of the intervention [20]. They provide a structured approach to the collection and organization of key background information, and a means to develop a snapshot or picture of the existing evidence base [16,17,20]. Furthermore, the findings of scoping reviews can be used to inform systematic reviews.

## Methods

**Figure 1:**
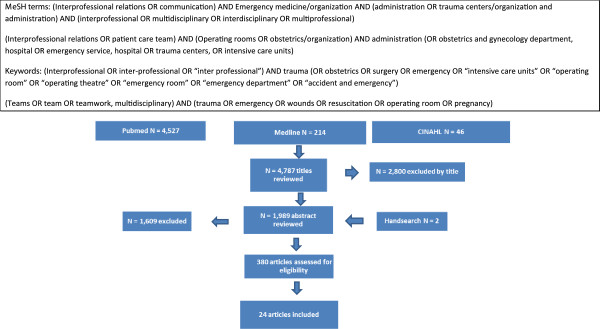
Search terms and study selection process.

The following sources were searched for results of interprofessional teamworking in the trauma setting published in peer-reviewed journals from January 2000 to April 2013: MEDLINE (via OVID, using keywords and MeSH in OVID), PubMed (via NCBI using MeSH), and CINAHL (Figure 1 shows the combination of search terms and the study selection process). Only articles in English were considered. A hand search was conducted by reviewing the reference lists of relevant articles. In line with the recommendation proposed by Levac *et al*. [20], the inclusion and exclusion criteria were discussed at the beginning of the scoping process, and the search strategy was refined as the abstracts and articles were retrieved from the search. Abstracts were reviewed independently by two researchers, and frequent discussions took place in order to resolve any uncertainties with regard to the articles that should be included in the full article review. The full articles were reviewed by two independent reviewers in order to determine those to be included. A spreadsheet was created to chart relevant data (data collection categories included author, setting, study aim and design/intervention, sample size, and results/outcome measures), to enable the identification of commonalities, themes, and gaps in the literature [17]. Eligible articles included in the review described the organization of teams in the trauma setting, team composition and structure, and evaluations of teamwork interventions.

## Results

In total, 24 published articles were identified for inclusion in the review. Studies were both descriptive and evaluative (Table 1, Table 2) and could be categorized into three main areas: 1) descriptions of the organization of teams in the trauma setting; 2) descriptions of team composition and structure; and 3) evaluation of team work interventions. Within each area a number of themes were identified, and each of these themes is discussed below.

**Table 1:** Descriptive studies

**Author**	**Setting**	**Research aim/question**	**Design**	**Sample size**	**Findings**
Anderson & Talsma, 2011 [31]	OR	To determine how the operating room staffing of two surgical specialties compare in terms of social network variables	Examination of staffing data, using social network analysis	Data were collected from 4,356 general surgery cases and 1,645 neurosurgery cases	Team coreness was associated with length of case. Procedures starting later in the day were less likely to be staffed by a team with a high number of core members. RNs constituted the majority of core interdisciplinary team members
Arakelian et al., 2011 [25]	OR	To study how organized surgical team members and their leaders understood operating room efficiency		11 (9 team members, 2 team leaders)	Seven ways of understanding operating room efficiency were identified
Cassera et al., 2009 [29]	OR	Team size and effect on team performance	Retrospective case review	360 laparoscopic procedures	Mean team size was eight members. Surgeons and anesthesiologists were constant team members, while the OR nurses changed more than once in each procedure. Surgery complexity and team size significantly affected PT; adding one person to the team increased PT by 15.4 minutes
Cole & Crichton, 2005 [28]	ED	To explore the culture of a trauma team in relation to the influence that human factors have over its performance	Ethnography/interviews/observation	6 periods of observation and 11 semi-structured interviews	Leadership, role competence, conflict, communication, environment, and patient status all influenced the culture of the trauma team
Creswick et al., 2009 [32]	ED	To use social network analysis to measure communication patterns and staff interactions within an ED	Social network survey and social network analysis	103 ED staff	Communication across the ED could be clearly understood in terms of three professional groups; interactions between individuals occurred mainly within professional groups
Gillespie et al., 2010 [24]	OR	To extend understanding of the organizational and individual factors that influence teamwork in surgery	Grounded theory/interviews	16 OR staff (surgeons, anesthetists and nurses)	Three themes described interdisciplinary teamwork practice: 1) contribution of interdisciplinary diversity to complex interpersonal relations; 2) the influence of the organization; 3) education
Leach et al., 2009 [21]	OR	To describe the nature of surgical teams and how they perform in the OR, in otder to contribute to a broader knowledge about high-performing teams and high-reliability teams in healthcare settings	Qualitative/observational study and interviews	Field observations of 10 high complexity surgeries	Coordination type and degree of independent and interdependent coordination varied between the observed stages (n = 7) of the surgical process. Teams were mainly ad hoc. Teams were challenged by ‘hand-offs’ and role demands that interfered with the adaptive capacity of the team
				Surgeries and face-to-face interview with 26 team members	
Lingard et al., 2004 [23]	ICU	An exploration of the interaction between ICU team members	Focus groups	Seven focus groups, each lasting 1 hour, with nurses, resident groups, and intensivist groups	Perception of ‘ownership’ and the process of ‘trade’ were mechanisms by which team collaboration was achieved or undermined
Sakran et al., 2012 [27]	Level 1 trauma center	To evaluate the relationship between the perception of leadership ability and efficiency of trauma patient care	Prospective observational study using a Campbell Leadership Descriptor Survey tool	81 leadership surveys collected from 22 separate trauma patient resuscitation encounters	The trauma teams perception of leadership was associated positively with clinical efficiency
Sarcevic et al., 2011 [26]	ED	To identify leadership structures and the effects of cross-disciplinary leadership on trauma teamwork	Ethnography/observation/interviews	100 hours of observations at 60 trauma resuscitation events, and 16 interviews with team members	Identified five leadership structures under two categories: 1) solo decision-making and intervening models within intradisciplinary leadership; and 2) intervening, parallel, and collaborative models within cross-disciplinary leadership
Weller et al., 2008 [22]	OR	To improve patient safety by gaining an understanding of OR team interaction, and to identify strategies to improve the effectiveness of the anesthesia team	Qualitative study/interviews following simulation of anesthesia crises	20 telephone interviews	Limited understanding of roles and capabilities of team members, differing perceptions of roles and responsibilities, limited information-sharing between team members, and limited input among team members in decision-making
Zheng et al., 2012 [30]	OD	Effect of surgical team size on team performance	Review of general surgery procedures over a 1 year period	Reviewed records of 587 procedures	Eight members per team on average. Half the team members were nurses. Surgery complexity and team size significantly affected PT; the addition of one team member predicted a 7 minute increase in PT

OR, operating room; ED, Emergency department; ICU, intensive care unit; OD, operating department; RRT, rapid response team; LM, leadership and management; PT, procedure time.

**Table 2:** Evaluative studies

**Author**	**Setting**	**Research aim/question/hypothesis**	**Design**	**Intervention**	**Sample size**	**Outcome measures**	**Tools**	**Results**
Bleakley et al., 2006 [34]	OT	Whether a sustained complex educational intervention would result in incremental, longitudinal improvement in attitudes and values towards interprofessional teamwork	Quasi-experimental, pre-test and post- test measures (findings from round 2 of the intervention)	Three strands: 1) data-driven iterative education in human factors; 2) establishment of a local, reactive ‘close call’ incident-reporting system; 3) team self-review (briefing and debriefing across all teams)	All general, trauma, and orthopedic theaters within one teaching hospital and two small satellite units	Teamwork climate	Validated SAQ	Intervention group had a higher aggregate teamwork climate score
Bleakley et al., 2012 [35]	OT	Whether a sustained complex educational intervention would result in incremental, longitudinal improvement in attitudes and values towards interprofessional teamwork	Pre-interventino and post-intervention (findings from round 3 of the intervention)	Three strands: 1) data-driven iterative education in human factors; 2) establishment of a local, reactive ‘close call’ incident = reporting system ; 3) team self-review (briefing and debriefing across all teams	All general, trauma, and orthopedic theaters within one UK teaching hospital and two small satellite units	Teamwork climate	Validated SAQ	Mean ‘teamwork climate’ scores improved incrementally and significantly
Brock et al., 2013 [40]	Medical, nursing, pharmacy and physician assistant students at one university	For students to acquire effective interprofessional team communication skills	Pre-test/post-test	Didactic instruction on patient safety and TeamSTEPPS communication skills. Students divided into IP teams for three simulated exercises and debriefing (observer/participant role) (4 hour training block)	149 students completed pre-test and post-test assessments	Attitudes towards team communication; attitude/knowledge/motivation/utility/SE towards IP skills; key communication behaviors; understanding; program evaluation	Validated TeamSTEPPS TAQ, AMUSE, self-report/Likert scale	Significant differences across all outcome measures
Capella et al., 2010 [41]	Level 1 trauma center	Does trauma team training improve team behaviors in the trauma room? If so, does improved teamwork lead to more efficiency in the trauma room and improved clinical outcomes?	Pre-training/post-training intervention design	2 hour didactic instruction (roles, responsibilities, TeamSTEPPS essentials (that is, communication tools)) and simulation in a learning center/simulation laboratory	33 trauma resuscitations pre-training, 40 post-training	Assessment of team performance; clinical outcome and clinical timing data	Validated TPOT	Significant improvement in all teamwork domains. Significant improvements in some clinical timing/outcome measures
Catchpole et al., 2010 [33]	Surgery (maxillofacial, vascular and neurosurgery)	The effects of aviation-style training on three surgical teams from different specialties	Prospective study before and after an intervention	1 to 2 days class-based series of interactive modules (including teamwork, communication, leadership, basic cognition, SA, decision-making, briefing, and debriefing) followed by team coaching (value of briefing/debriefing)	112 operations (51 before and 61 after the intervention)	Attitudes to safety and cultural context. Frequency of pre-list briefings, pre-incision time-outs/stop checks, post-case debriefing, and dimensions of team skills	SAQ; structured observations and validated NOTECHS method to classify team skills	Significantly more briefings, debriefings, and stop checks. No improvement in teamwork
Mayer et al., 2011 [36]	Pediatric and surgical ICU’s	Implementation of TeamSTEPPS	Pre-training/post-training intervention design	Change team trained/coached front-line staff, comprising 2.5 hour training sessions and group training in practice (ad hoc rather than intact teams)	12 attending physicians, 157 nurses, 90 respiratory therapists	Staff interviews, observations of teamwork, clinical timing data, clinical infection data, perception of safety culture, strengths/weaknesses of the unit, job satisfaction	TENTS, EOS, HSOPC, NDNQI	Significant improvements in team performance/perception of teamwork (12 month follow-up). Significant decrease in clinical timing
Miller et al., 2012 [37]	Level 1 trauma center/ academic tertiary care center	An ISTSP could be implemented in the ED and this would improve teamwork and communication in the clinical setting	Pre-training/post-training intervention design involving all members of the trauma team	Standardized lecture that specified roles, responsibilities, order of tasks, andposition in resuscitation area followed by simulation (ad hoc teams)	39 real trauma activations observed	Teamwork and communication	Validated clinical teamwork scale	Teamwork and communication improved, but effect not sustained after the program
Nielson et al., 2007 [38]	Obstetrics	To evaluate the effect of teamwork training on the occurrence of adverse outcomes and process of care in labor and delivery	Cluster randomized controlled trial	Instructor training session: standardized teamwork training curriculum based on CRM, which emphasized communication and team structure. Instructor created ad hoc workplace teams	1,307 personnel trained and 28,536 deliveries analyzed	Adverse maternal/neonatal outcomes; clinical process measures	Adverse outcome index	Training had no significant effect
Wallin et al., 2007 [43]	Trauma	To evaluate the outcome of a CRM target-focused instructional strategy on team behavior and attitude	Prospective study	Simulation	15 medical students; observations of 8 trauma scenarios in simulation classroom	Behavior performance, team attitude	Structured observation schedule	Improvement in observed team behavior. No attitude change
Weaver et al., 2010 [44]	OR	Does TeamSTEPPS training meaningfully affect teamwork behavior in OR teams? Does this teamwork positively affect important outcomes such as patient safety culture?	Mixed model design with one between-groups factor and two within-groups factors	TeamSTEPPS training curricula, including a 4 hour didactic session (competency-based), including interactive role-playing activities	Three surgeons and their teams	Trainee reactions, trainee learning, behavior on the job, results (degree to which training affected safety/quality)	Questionnaire survey; TeamSTEPPS learning benchmark test; CATS observation tool; HSOPS; ORMQ	Positive results at all levels of evaluation
Wolf et al., 2010 [42]	OR	MTT has been touted as a way to improve teamwork and patient safety in the OR	Post-training data collection	1 day didactic modules for all staff, with video and role-play. Topics included human factors, communication, fatigue recognition, briefing/debriefing training	4,863 MTT debriefings analyzed	Team functioning, case delays, case scores	Debriefing/briefing checklist	Case delays decreased and case scores increased; sustained at 24 months. Improved perception of patient safety and teamwork
Wisborg et al., 2006 [39]	Trauma	To describe a team intervention and assess the feasibility of the intervention	Pre-training/post- training; intervention design	3.5 hour didactic session (teamwork/cooperation/ communication skills) and practical training session for all trauma team members in practice using simulation and debriefing	28 Norwegian hospitals and 2,860 trauma team members participated in the training	Evaluation of experience	Questionnaire	High perception of learning

AMUSE, Attitude, Motivation, Teamwork, Self Efficacy; CRM, crew resource management; ED, emergency department; EOS, Employee Opinion Survey; HSOPC, Hospital Survey on Patient Safety Culture; ICU, Intensive Care Unit; IP, interprofessional; ISTS, *In situ* Trauma Simulation; MTT, medical team training; NDNQI, National Database of Nursing Quality Indicators; NOTECHS, no technical skills; OR, operating room; OT, operating theater; POT, Trauma Team Performance Observation Tool; SAQ, Safety Attitudes Questionnaire; SA, situational awareness; SE, self efficacy; TAQ, Teamwork Attitude Questionnaire; TeamSTEPPS, Team Strategies and Tools to Enhance Performance and Patient Safety; TENTS, Team Evaluation of Non-technical skills.

### Descriptions of the organization of teams in trauma settings

#### Interactions between team members

Four studies [21-24], used qualitative methods to explore interactions between team members. Teams were described as dynamic/fluid and involving seven stages (many of which occur in a parallel fashion) on a continuum from coordinated independent behaviors through to coordinated interdependent behaviors [21], with the stages ebbing and flowing depending on patient need [21]. Professional independence, although at times limiting interprofessional collaboration, enabled individuals to work cohesively together under pressure. In situations where team members were unknown to one another, the coordination of activities within a professional group contributed to team efficiency and performance [24].

Teams were found to be primarily *ad hoc* in nature [21]. The changing dynamics of a team was seen to influence its adaptive capacity. For example, high turnover and short-term involvement of team members hindered team performance. Ability to anticipate team members’ needs, adaptive capacity [21], ability of the physician to create a good working environment [21], work space [21], team familiarity with procedures [21,22], and the right mix of technical competency [21-24] were all factors identified as important for effective team working. Organizational processes and management had a potentially negative influence on teamwork [21-24]. Valued commodities (including technical skills and knowledge, equipment, clinical territory) were identified as forming the basis of negotiation or exchange in interprofessional interactions and facilitating collaboration [23].

#### Leadership

Four studies [25-28] used qualitative methods to explore how leadership influences teamworking. The role of the team leader has been described as pivotal for effective team function, as they have responsibility for team members and the direction of all team activity [28]. Sakran *et al*. [27] found a positive relationship between team efficiency and the perception of leadership by team members. Teams directed by surgeons who were perceived as having low leadership ability took significantly longer to complete the key steps in initial trauma patient evaluation. Team leaders who had positive effects on performance were described as encouraging and as motivating team members through positive behavior and feedback. Leaders who used power and authority had negative effects on performance [27]. Five leadership structures during trauma resuscitation were identified by Sarcevic *et al*. [26]: intradisciplinary leadership, solo decision-making, cross-disciplinary leadership, shared decision-making, and collaborative decision-making. Where leadership was intradisciplinary and decisions were made by a single team leader, information exchange and teamwork were facilitated, because team members had a clear understanding of who was the team leader. However, negative effects included an increased likelihood of performing unnecessary procedures. By contrast, cross-disciplinary leadership and collaborative decision-making had positive effects on overall team performance. Resuscitation events ended with positive feelings shared between leaders and team members, and conflicts were less likely to occur.

### Descriptions of team composition and structure

#### Stability of team members

Two studies [29,30] examined the effect of surgical team size on team performance, and emphasized the importance of the stability of the team members. A retrospective case review of general laparoscopic procedures (n = 399) undertaken over a 2 year period [29] identified that although anesthesiologists and surgeons normally stayed for the entire surgery, nurses often did not, because of breaks and shift changes. Most procedures were assisted by two scrub nurses working in succession. However, nearly 25% of the procedures were assisted by between three and five nurses. The majority of procedures were also attended by two circulating nurses working in quick succession. However, nearly 25% of the procedures were attended by three circulating nurses. In extraordinarily long procedures (5%), four circulating nurses attended. The authors suggested that complete involvement with a procedure enables a surgeon and anesthesiologist to develop a comprehensive shared mental model regarding tasks and goals. High turnover and short-term involvement of other team members requires better communication strategies to keep them updated with the current state of procedures. The authors further suggested that high turnover hinders team performance and leads to distraction and loss of focus. Their results confirmed that when team size was increased, the procedure time (PT) was prolonged, independent of other factors, including surgical complexity. Adding one additional team member to a surgical team predicted a 15.4 minute increase in PT. Recommendations included the need to develop strategies to construct the team inside the OR without constantly changing the composition, especially for nurses in a team. Similar work was undertaken by Zheng *et al*. [30], who reviewed the records of 640 procedures. These researchers identified that a change in one team member was associated with a 7 minute increase in PT. They emphasized the importance of maintaining the stability of core team members, and the implementation of measures to reinforce the quality of communication between members when role changes occur.

#### Core team members

Two studies [31,32] used social network analysis to understand and characterize staffing patterns. The extent to which core team members worked together (or ‘team coreness’) was influenced by professional group and affected by length of procedure. Creswick *et al*. [32] reported that despite the emergency department often being construed as one team, communication could be better understood in terms of individual professional groups. Individuals in this study were found to rely heavily on their own professional group to solve work-related problems. Anderson and Talsma [31], also using social network analysis, explored staffing in the OR. Their findings showed that the longer the surgery, the more likely that the OR would be staffed with core team members. Furthermore, cases that started later in the day were less likely to be staffed by core team members, and longer cases were more likely to start earlier in the day. The longer the case, the more likely core members were involved. Anesthesia residents and registered nurse (RN) anesthetists were not members of core groups. RNs accounted for two to three times the percentage of each core group membership. The authors suggested that, based on their results, core team members appear to be assigned to work on the longer and more complex procedures.

### Evaluation of teamwork interventions

#### Activities in practice

Seven studies, [33-39] comprised interventions that, in addition to didactic instruction, involved a range of activities in practice (for example, simulation [37-39] coaching, [33-39] team self-review/reporting system [34,35], and group training [30]). An array of topics (attitude to safety, team climate, team performance, roles and responsibilities, situation awareness, co-operation, debriefing), were covered during didactic instruction. CRM formed the basis of interventions in three studies [33,36,38]. Training in practice involved both intact [33-36] and *ad hoc* teams (that is, teams put together for the purpose of the research) [37-39]. Outcomes measured included attitude to safety, frequency of briefings, dimensions of team skills, [33] team climate [34,35], teamwork, clinical timing and outcome data [36-38], teamwork and communication [37], and evaluation of learning experience [38,39]. Findings were generally positive. Only two studies [35,36] reported on the long-term effect of the intervention. Meyer *et al*. [36] reported significant improvement in team performance and perceptions of teamwork and significant decrease in clinical timings at 12 months. Mean ‘teamwork’ climate scores were found by Bleakley *et al*. [35] to improve incrementally and significantly over a 4 year period.

#### Activities in the classroom setting

Five studies [40-44] comprised interventions delivered in the classroom setting. As well as didactic instruction, three of these studies [40,41,43] involved participants in simulation. Patient safety, Team Strategies and Tools to Enhance Performance and Patient Safety (TeamSTEPPS) communication skills, roles and responsibility, human resources, and briefing and debriefing were topics included in didactic instruction. CRM formed the basis of interventions in all of these studies. Apart from work by Weaver *et al*. [44], interventions were delivered to *ad hoc* teams. Outcome measures included attitudes [40-43], team performance [41-43], team function case delays and case scores [42], and learning behavior [44]. Only Wolf *et al*. [42] reported a sustained effect at 24 months.

## Discussion

The studies included in this review were undertaken in developed countries and therefore, the findings may not be applicable to other settings. The limitation of the descriptive studies is that they describe changes in the attitudes, values, and perceptions of practitioners, as opposed to changes in their behavior and performance or in outcomes. Furthermore, several of the studies had only small numbers of participants in single location settings and so findings may be different in other areas of trauma care. Evaluative studies have a number of weaknesses, including small sample size, short follow-up period, and lack of control. Very few studies used validated measures and little information about human resources was provided. Extraneous factors make it difficult to identify a causal relationship between the teamwork intervention and the result.

It is evident from the descriptive literature included in this review that a number of attributes characterize effective interprofessional teamwork in the trauma setting. Firstly, studies that described the organization of trauma teams and ‘interactions between team members’ depicted team processes as fluid in nature, and outcomes as accomplished through the interactive effort of all professionals involved. Team functioning is described as a continuum from coordinated independent behaviors through to coordinated interdependent behaviors [21], and are dependent upon patient need. Responsibilities are described as ‘shared’ with a high level of communication and collective decision-making. Secondly, studies detailing team composition and structure, and specifically those within the theme ‘maintaining team stability,’ described high-performing interprofessional teams as having a shared mental model [29]; that is, team members were familiar with one another’s roles and responsibilities; they were able to anticipate the needs of team members, and had a high level of adaptive capacity. Adaptive capacity is affected by staff turnover, which in turn can affect team performance and performance time. Thirdly, the theme ‘leadership’, also within the descriptive literature that describes team organization, refers to leaders as being pivotal for the effective coordination of team members’ contributions. Effective teams were those in which leaders made collaborative decisions across disciplines [26].

Many of the interventions and outcome measures used in evaluative studies are based on CRM. Studies within the two themes that arose from this literature (‘activities in practice’ and ‘activities in the classroom setting’) comprised didactic instruction, which covered a range of topics including leadership and decision-making [33], briefing and debriefing [34,35], and patient safety and communication skills [40]. Several studies focused upon roles and responsibilities [38,42,44], and order of tasks [37]. Practical training primarily involved the *ad hoc* structuring of teams by researchers, trainers, and managers, who were put together to work on simulated cases in practice or in the classroom environment. Although the effects on outcome measures, including teamwork climate [34,35], attitude to team communication and interprofessional skills [40], teamwork performance [36], clinical timing data [36,41,42] ,and observed team behavior [43,44], were positive, very few studies reported on whether or not these effects have been sustained. Furthermore, little or no information was provided across these studies on group processes or dynamics, group structure, and how teams were led, which are of major relevance to patient safety.

Researchers in the UK examining interprofessional teamwork across community rehabilitation and intermediate care settings [14] have similarly identified a paucity of information on interdisciplinary team structure and processes in RCTs. In line with the findings from the current review, other researchers identified communication between and interactive effort of all team members as a characteristic of good interprofessional team functioning [4]. However, by contrast, team functioning was not identified as a continuum from coordinated independent behaviors through to coordinated interdependent behaviors, and a shared mental model between team members was not recognized as a characteristic of good interprofessional teamworking. This is perhaps not surprising given that the trauma setting involves the management of complex patients by specialized teams in a dynamic environment, and the UK research involved teams in community rehabilitation and intermediate care settings. These services were not high risk (nor was the environment dynamic), but were rather designed to provide rehabilitation and care for older people.

## Conclusion

Medical errors occur primarily as a result of system failure rather than the action of an individual. Such errors are grounded in shared activities, involving teamwork and communication, as opposed to profession-specific technical expertise [29]. Therefore, in order to improve patient safety, changes in teamwork practice are crucially important. This is reinforced by the findings of the descriptive studies reported in this review: effective interprofessional teamwork was seen as a continuum from coordinated independent behavior through to coordinated interdependent behavior, team members had a shared mental model, and leaders made collaborative decisions across disciplines. Many of the evaluative studies reviewed placed great emphasis on specialized roles and individual tasks and activities. This reflects a multiprofessional model as opposed to an interprofessional model of teamwork. Although it is vital that team members have the knowledge and skills to perform the role tasks, it is also important that research should focus on the interactions and processes rooted within these tasks.

Some of the characteristics of high-performing interprofessional teams described in this review are also evident in effective teams in the community rehabilitation and intermediate care setting. These characteristics may well be pertinent to other settings, and so provide a useful foundation for future investigations.

These findings should be borne in mind by those involved in team development, such as human resource practitioners and managers. Team development activities should ensure that team members value the importance of shared responsibility, communication, and collective decision-making, and have a good understanding of the roles of team members.

## Competing interests

The authors declare that they have no competing interests.

## Authors’ contributions

MC, SN, and DD contributed towards the conception and design of the study, database searches, acquisition of the data, and analysis and interpretation of papers included in the review. MC drafted the manuscript. SN and DD provided critical revisions. All authors read and approved the final manuscript.
